# New species and records of *Charisius* Champion from Mexico and Central America (Coleoptera, Tenebrionidae, Alleculinae)

**DOI:** 10.3897/zookeys.415.6794

**Published:** 2014-06-12

**Authors:** J. M. Campbell

**Affiliations:** 1 Canadian National Collection of Insects, Arachnids and Nematodes, Agriculture and Agri-Food Canada, Ottawa, Ontario, K1A 0C6

**Keywords:** Coleoptera, Tenebrionidae, Alleculinae, *Narses*, *Charisius*, Mexico, Central America, Systematics, New synonymy, New species, New Combination

## Abstract

The species of the genus *Charisius* Champion, from Mexico and Central America are reviewed. The flightless genus *Narses* Champion, with one included species, *N. subalatus* Champion, is placed in synonymy with the genus *Charisius*. Four new species are described and illustrated, *C. granulatus* and *C. punctatus* (from Guatemala) and *C. apterus* and *C. howdenorum* (from Mexico). *Charisius subalatus* (Champion) is redescribed and illustrated. The species *C. interstitialis* Champion is placed in synonymy with *C. zunilensis* Champion. The genus is redescribed to include the four new species and *N. subalatus*. New distributional records are presented for all other species of the genus and a revised key is presented for identification of all the species of the genus.

## Introduction

The purpose of this paper is to record and describe the new species and distributional records of the genus *Charisius* Champion that have accumulated since my previous revision of the genus. I have placed the genus *Narses* Champion in synonymy with *Charisius* and have redescribed its only included species, *Narses subalatus*. The classification and arrangement of the species described in the earlier paper is followed except for the addition of the subalatus group to include the two flightless species. The four new species and *Narses subalatus* are described and illustrated.

## Methods

All measurements were made with an ocular micrometer mounted in a Leitz stereoscopic microscope. Measurements were made of the overall length from the anterior margin of the labrum to the apex of the elytra; the ocular index (OI) of both males and females; the lengths of the third and fourth antennomeres; the length and width of the tenth antennomere and the pronotal index (PI). The photographs were made with a Leica Digital DC500 Imaging Workstation using Zerene Stacker software and retouched with Adobe Photoshop software.

The terminology used in this paper is that recommended by Lawrence et al (2010). Other terms were described in my previous paper (1965) except the terms ocular index and pronotal index. The ocular index is the ratio of the distance between the eyes to the greatest distance across the eyes times 100. The pronotal index is the measurement of the width of the pronotum at its widest divided by the length of the pronotum along the midline times 100. My previous paper (1965) should be consulted for full descriptions and illustrations of all the previously described species.

Material was examined from the following collections:

AMNH American Museum of Natural History, New York, NY.

CASC California Academy of Sciences, San Francisco, CA

CMNC Canadian Museum of Nature, Alymer, Quebec, Canada

CNCI Canadian National Collection of Insects, Ottawa, Ontario, Canada

EGRC E. G. Riley Collection, Texas A & M University, College Station, TX.

JEWC J. E. Wappes Collection, San Antonio, TX.

USNM United States National Museum, Washington, DC.

UNMO University of Montana, Bozeman, MON.

Holotypes are deposited in the Canadian National Collection of Insects, Ottawa (CNCI), and the Collection of the University of Montana (UMON)

The dorsal habitus of all species are illustrated to show the various color patterns of each species and the male terminalia of all the new species and *Charisius subalatus* are illustrated with photographs.

## Systematics

### Charisius

Champion

http://species-id.net/wiki/Charisius

Charisius Champion (1888: 421); Champion (1893: 565); Campbell (1965: 43).
Narses Champion (1888: 423). Syn. n.

#### Description.

Body narrowly elongate (Figs 1–6, 9–12) (broader in subalatus group, Figs 7–8); glabrous dorsally; color ranging from light to dark reddish-brown, often with yellow and/or piceous markings on elytra. Surface varying from smooth, shining, with very fine microsculpture (visible only under high magnification) to dull with dense, moderately fine, visible microsculpture. Length 6-13 mm.

**Figures 1–6.**
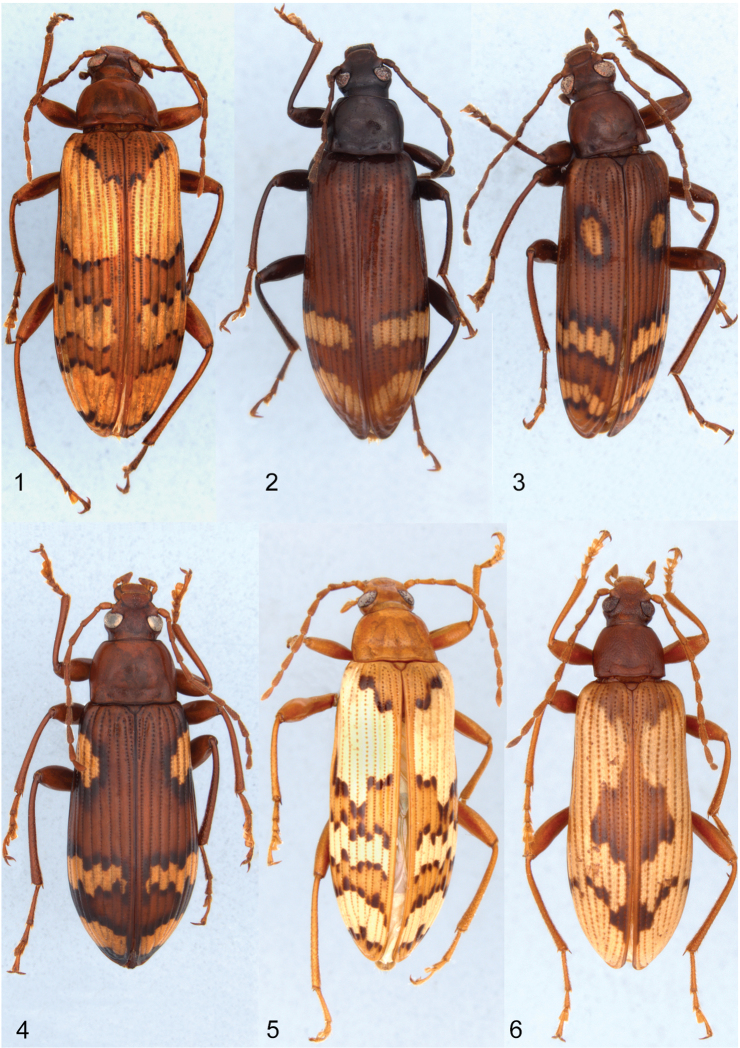
Dorsal habitus of species of *Charisius*: **1** *Charisius fasciatus*, from Tinijapa, 8 mi NE San Cristobal de las Casas, Chiapas, Mexico **2** *Charisius fasciatus*, from 8 km NE San Lorenzo, Zacapa, Guatemala **3** *Charisius fasciatus*, from Santa María, Quezaltenango, Guatemala **4** *Charisius picturatus*, from Route 190, 33.0 miles NW Oaxaca, Oaxaca, Mexico **5** *Charisius mexicanus*, from 8 km S Suchixtepec, Oaxaca, Mexico **6** *Charisius granulatus*, from 29 km N San Augustín, El Progresso, Guatemala.

**Figures 7–12.**
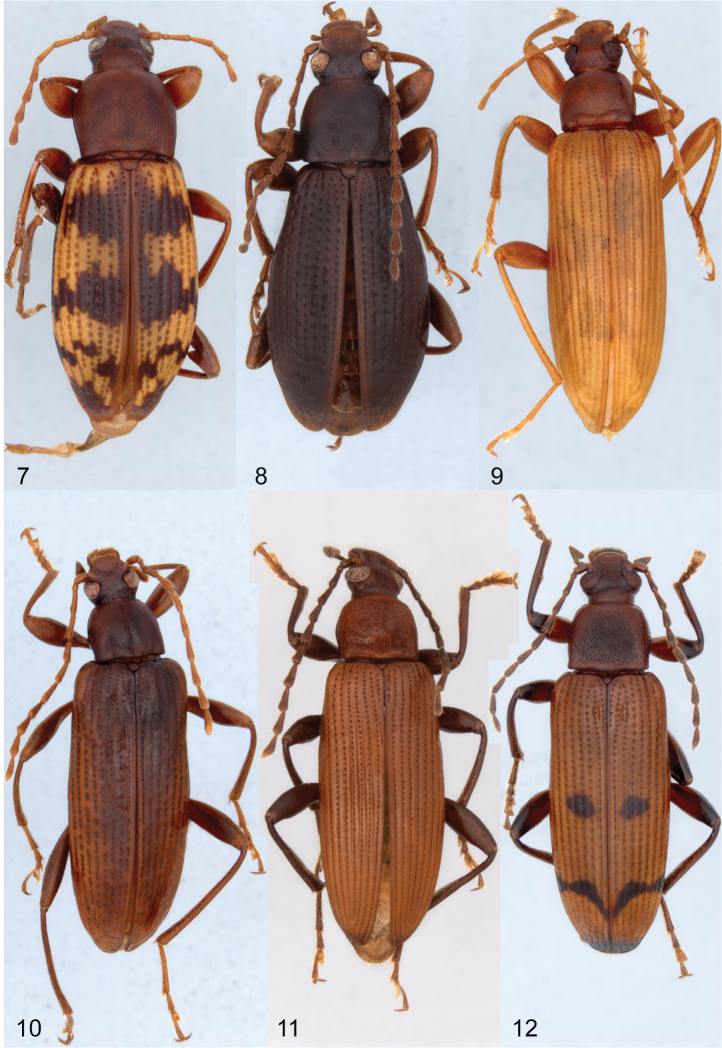
Dorsal habitus of species of *Charisius*. **7** *Charisius apterus*, from 2 mi. S Cerro Pelon, Oaxaca, Mexico **8** *Charisius subalatus*, from Miramundo, Jalapa, Guatemala **9** *Charisius zunilensis*, from 6 mi N San Lorenzo, Zacapa, Guatemala **10** *Charisius howdenorum*, from Tinijapa, 8 mi. NE San Cristobal de las Casas, Chiapas, Mexico **11** *Charisius salvini*, from Finca Florencia, Sacatepequez, Guatemala **12** *Charisius punctatus*, from San Lorenzo, Zacapa, Guatemala.

Head moderately sparsely to moderately densely, evenly punctate on vertex; punctures separated by average distance equal to or slightly greater than diameter of a puncture. Eyes moderate in size, separated by distances ranging from equal to or slightly greater than diameter of eye (OI ranging from 30 to 47); with distinct, well-developed nuchal-constriction (Figs 2, 4). Maxillary palpus (Fig. 16) with apical segment broadly securiform; apex subequal in length to outer side; mandible with apex shallowly notched medially. Antennae narrowly elongate (Figs 1–6); antennomere 2 very short, antennomere 3 much longer than 2, slightly shorter than to slightly longer than 4; 4–10 each elongate, at least two times longer than wide; sides only slightly widened from base to apex; antennal sensoriae small, visible only under high magnification, evenly distributed on segments 4–11.

**Figures 13–15.**
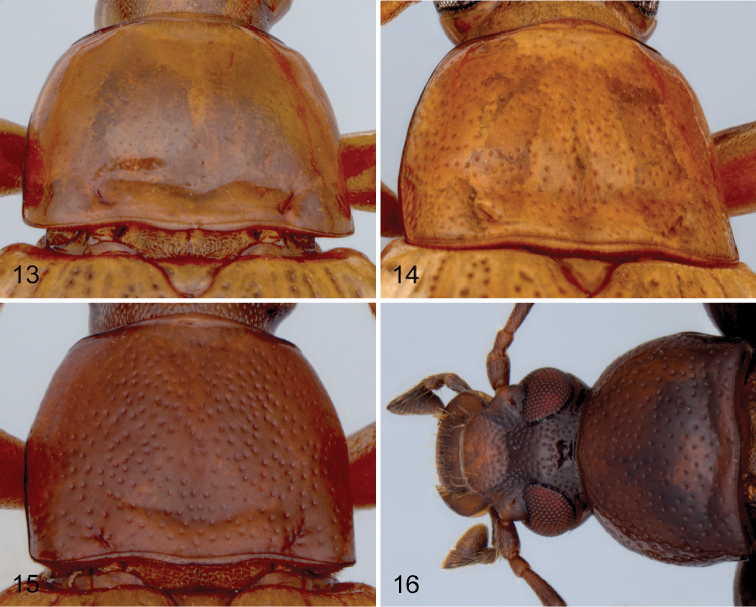
Pronotum of species of *Charisius*: **13** *Charisius fasciatus* **14** *Charisius mexicanus* **15** *Charisius granulatus* **16** Head and pronotum of *Charisius salvini*.

**Figures 17–20.**
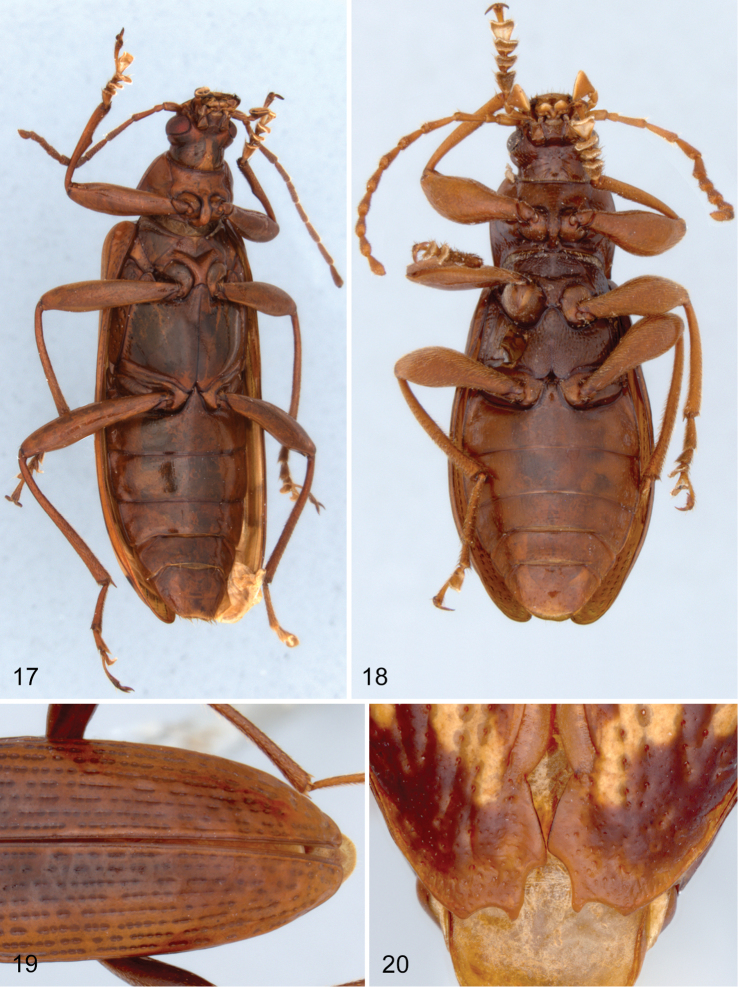
Ventral view of species of *Charisius*: **17** *Charisius fasciatus* **18** *Charisius subapterus* **19** elytral striae of *Charisius howdenorum* **20** apex of female elytra of *Charisius apterus*.

Pronotum with base distinctly narrower than base of elytra (Figs 1–10); sides variable, ranging from evenly narrowed from base to near apex to widest near middle and curved both anteriad and posteriad; width greater than length (PI ranging from 67 to 95); anterior margin truncate to slightly convex; anterior angles distinct, narrowly rounded. Basal foveae small, moderately deeply impressed, connected across base of pronotum by distinct transverse, prebasal groove. Prosternum elongate (Figs 17–18), horizontal anteriad of procoxae, prosternal process evenly rounded, abruptly declivous anteriad and posteriad of procoxae. Mesoventrite elongate (Fig. 17), distinctly more elongate than mesocoxal cavities except in species of subalatus group (Fig. 18); with shallow to moderately deep, V-shaped mesoventral cavity; intercoxal process gradually sloped to prosternum. Metaventrite with surface finely, sparsely to moderately coarsely punctate; disc distinctly more elongate between coxae than length of mesocoxal cavities (except in species of subalatus group). Third and fourth segments of anterior and intermediate tarsi and penultimate segment of posterior tarsi lobed ventrally, in addition, basal two segments of protarsus lobed ventrally in male (except *Charisius salvini*).

Elytra elongate (Figs 1–6, 9–12); sides parallel for basal half; then evenly narrowed to apex except *Charisius apterus* and *Charisius subalatus* (Figs 7–8); striae moderately shallowly impressed near base, becoming more deeply impressed approaching apex (striae unimpressed between strial punctures in *Charisius howdenorum* (Fig. 19); strial interstices usually convex or rarely flat; impunctate or with a row of fine, median punctures visible only under high magnification. Elytral epipleurae ending just before apex of elytra; evenly arched from base to apex. Ventrites finely, sparsely punctate or impunctate.

**Figures 21–24.**
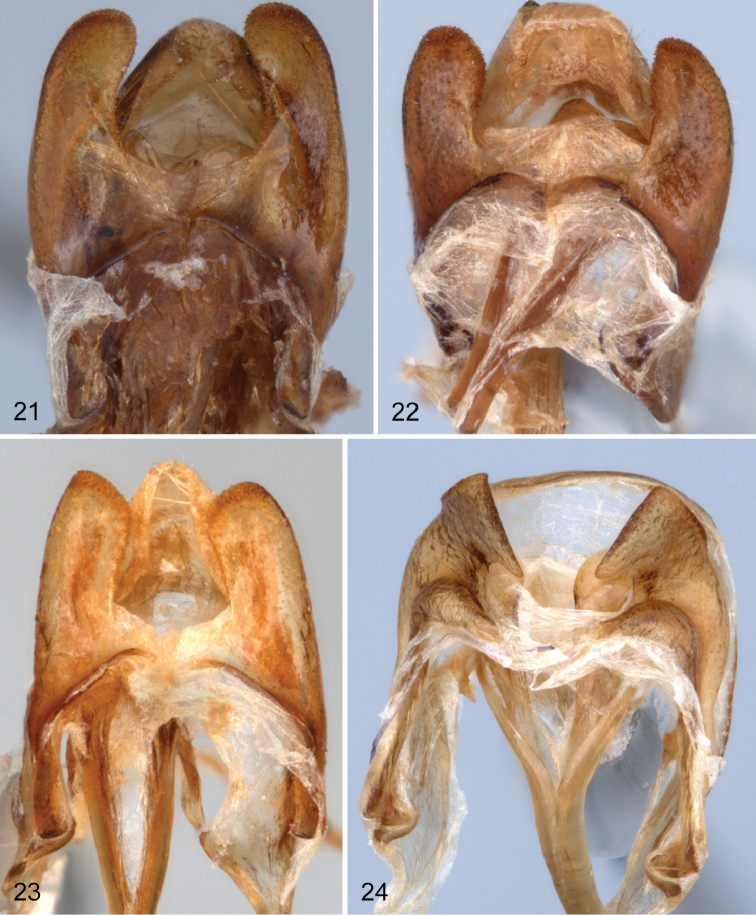
Ventral view of lobes of male eighth and ninth sterna of species of *Charisius*: **21** *Charisius granulatus* **22** *Charisius subalatus* **23** *Charisius howdenorum* **24** *Charisius punctatus*.

**Figures 25–28.**
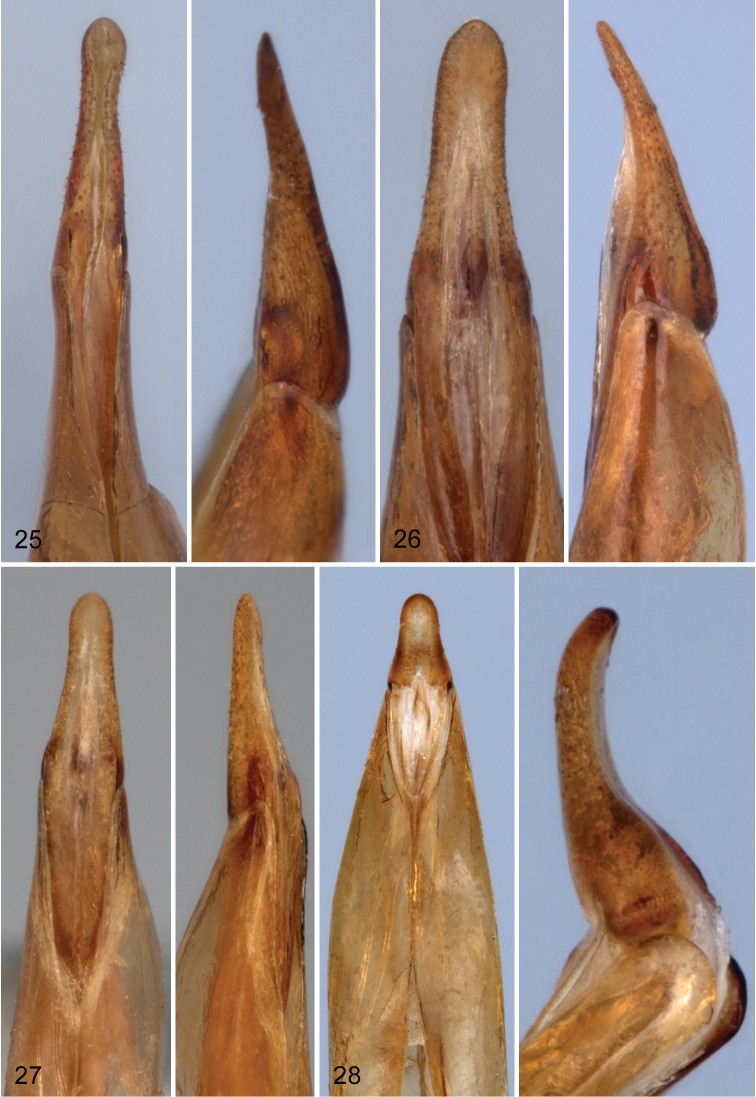
Apicale of aedeagus of species of *Charisius*; left, ventral view; right, lateral view **25** *Charisius granulatus* **26** *Charisius subalatus* **27** *Charisius howdenorum*; **28** *Charisius punctatus*.

Male: Eighth sternite divided into two large, well developed lobes (Figs 21–24); apex of each lobe appearing glabrous, actually bearing small, densely placed, dentiform setae which extend along inner margin to near base (visible only under high magnification). Ninth sternite bilobed; lobes small, not joined medially, reaching only to base of eighth sternal lobes. Apicale of aedeagus variable (Figs 25–28); ranging from 2.8 to 3.5 times as long as basale (apicale very short in salvini group, basale 6.9 to 7.9 times longer than apicale).

#### Type species.

I previously designated *Charisius fasciatus* Champion as the type species of the genus (Campbell 1965: 45). The type species of *Narses* is *Narses subalatus* Champion, by monotypy (Champion 1888: 423).

#### Remarks.

Champion (1888 and 1893) described the genus *Charisius* to include five species and the genus *Narses* to include only *Narses subalatus*. Subsequently, I revised the genus *Charisius* (1965) and included one additional species, *Charisius mexicanus*. In this paper, based on additional material, I have placed one species, *Charisius interstitialis* Champion, in synonymy with *Charisius zunilensis* Champion and have described an additional four new species. Species of *Charisius* occur in moderate to high elevations from central Mexico south to the highlands of Nicaragua. Records cited in this paper extend the known ranges of several species from Guatemala south to El Salvador and Honduras. One specimen of *Charisius salvini* was collected in Nicaragua.

Adults of *Charisius* are easily distinguished from all other Mexican and Central American members of the tenebrionid subfamily Alleculinae (Tribe Alleculini) by the combination of having the body glabrous dorsally; by the deep, prebasal transverse groove connecting the basal foveae of the pronotum; by having the elytral interstices impunctate except for a median row of minute punctures visible only under high magnification; by their elongate, almost filiform antennae, with the subapical antennomeres at least two times longer than wide; by their narrow and elongate shape (see Figs 1–12), and by the broadly securiform shape of the apical segments of the maxillary palpi (Fig. 16). Adults of seven of the ten species now known differ from those of all other species of Mexican and Central American Alleculina in having distinctive yellow and/or black markings on the elytra.

Champion distinguished the genus *Narses* from *Charisius* based only on the presence of reduced wings, stating that the genera are similar in all other respects. The discovery of a second flightless species, *Charisius apterus* and an examination of the male terminalia of all the species confirm that *Narses* and *Charisius* are congeneric.

I have retained the same species groups that I established in my previous paper except that I have added an additional group for the two flightless species.

#### Bionomics.

Adults of *Charisius* are found primarily throughout the rainy season. In Guatemala and southern Mexico the rainy season normally begins in March or April and the season ends usually in November. Adults are usually collected by beating dead leaves of thick, mixed vegetation. Apparently, there is no plant host specificity involved since I have collected specimens from the leaves of oaks, dead vines, tree ferns, and a number of different deciduous trees. I collected and reared one larva of *Charisius fasciatus* from rotting detritus from inside a hollow log. A pupa and one adult were collected from pupal cells about a meter above ground level in soft, decaying wood of a dead, standing oak.

### Fasciatus Group

Species of this group may be easily distinguished in having the wings fully developed, in having yellow, transverse markings on the elytra, by their larger size, in having the male anterior tibiae distinctly widened on the inner side near the middle (except *Charisius granulatus*), the male fifth visible ventrite not impressed medially and the apicale of the aedeagus elongate (Fig. 25). The basale of the aedeagus varies from 2.5 to 3.5 times longer than the apicale.

#### 1. Charisius fasciatus

Champion

http://species-id.net/wiki/Charisius_fasciatus

Figs 1–3 , 13 , 17

Charisius fasciatus Champion (1888: 421, pl. 19, Figs 12, 12a, 13); Campbell (1965: 67, Figs 3, 9, 15, 16).

##### Type.

Lectotype, male, Quiché Mountains, Guatemala (Campbell 1965: 47). The specimen is in the BMNH.

##### Distribution and records.

*Charisius fasciatus* was previously known from six specimens collected from the highlands of Guatemala. The following records extend the known range of this species into the state of Chiapas in southern Mexico, Honduras and El Salvador. It has been collected between the elevations of 1500 to 2750 meters.

EL SALVADOR: Cerro Verde, 2000 m, 1.V.1971, HF Howden (CMNC) 1.

GUATEMALA: El Progresso: 28-29 km N San Augustin, 7000–8500 ft, 17–21.IV.1990, JE Wappes (JEWC) 1. Jalapa: Miramundo, 8400 ft., 3.VII.1986, JMC (CNCI, JMCC) 4. Quetzaltenango: Santa María, Los Pirineos, 4500 ft, 15.VI. 1966, JMC (JMCC) 1; Santa María, 5,000 ft, 18.V.1966, JMC (JMCC) 1; Santa María, 6,000 ft, 10.VII.1965, JMC (JMCC) 1; 4 km W Santa María, 5000 ft, 27.III.1966, JMC (JMCC) 1; 3 km SE Zunil, Tzanjoyan, 2400 m, 1.XI.1965, JMC (JMCC) 2; 12 km SE Zunil, NW face Cerro Zunil, Fuentes Georginas, 2700 m, 17.VI.1993, R Anderson (CMNC) 1. Quiché: Nebaj, 6000 ft, 9.VIII.1947, C & P Vaurie (AMNH) 1. Suchitepéquez: 5 km S Santiago Atitlan, 1500 m, 13.IX.1965, JMC (JMC) 1; Zunilito, 2 km N Finca Colimas, 6000 ft, 28.IV.1966, JMC (JMCC) 2. Zacapa: 3 km NE San Lorenzo, Sierra de las Minas, 1800 m, 6.VII.1986, JMC (CNCI) 1; 6 km NE San Lorenzo, 6500 ft, 17.VI.1993, JMC (CNCI, JMCC) 2; 8 km NE San Lorenzo, Sierra de las Minas, 2100 m, 10.VII.1986 and 18.VII.1986, JMC (CNCI) 2.

HONDURAS: Francisco Morazán: 21.5 km N Tegucigalpa, PN La Tigra, 1950 m, 15.VIII–2.IX.1994, S&J Peck (CMNC) 1.

MEXICO: Chiapas: Cerro Huitepec (Pico), ca 5 km W San Cristobal de las Casas, 2750 m, 23.IX.1991, R Anderson (CMNC) 1; 5 km W San Cristobal, 3.V.1969, HF Howden (CMNC) 1; 6 km E San Cristobal, 9.V.1969, HF Howden (JMCC) 1; 8 km NE San Cristobal, 17.V.1969, HF Howden (CMNC) 1; 8 mi S Simojovel, 10.VI.1969, JMC (CNCI) 1; San Cristobal de las Casas, 26.VII.1969, LA Kelton (CNCI) 2; 8 mi NE San Cristobal, 28.VI.1969. JMC, (CNCI, JMCC) 5; same locality, 5.V.1969, HF Howden (CMNC) 2; 11 mi NE San Cristobal, 18.V.1969, HF Howden (CMNC) 3; Tinijapa, 8 mi NE San Cristobal 26.V.1969 and 18.V.1969, JMC (CNCI, JMCC) 12; 11 mi NE San Cristobal, 18.V.1969, HF Howden (CMNC) 2; Rte. 190, 16.8 mi SE Teopisca, 6700 ft., 2.IX.1967, Ball, TL Erwin, RE Leech (CNCI) 1.

##### Remarks.

This species is the most variable in color of any species of the genus. It closely resembles *Charisius picturatus*, *Charisius mexicanus*, and *Charisius granulatus* in some of its color patterns. No differences were noted in the structure of the aedeagus and the male seventh and eighth sternites between the males of these color forms. In my earlier revision I recognized two distinct color forms, one with three yellow, transverse bands across the elytra (from Totonicapán and the Quiché Mountains of Guatemala) and the second form with only two transverse bands (from Cerro Zunil, near Quezaltenango and Calderas, near Antigua in Guatemala). A long series of specimens from a number of areas near San Cristóbal de las Casas in Chiapas and a number of different localities in Guatemala are intermediate between these two previously recognized color forms, so I am unable to distinguish various color forms based on geographical variation.

Adults of *Charisius fasciatus* may be distinguished from those of *Charisius mexicanus* by differences in the structure of the male eighth sternal lobes and by the distinctly finer and sparser punctation of the pronotum (Figs 13, 14). Adults of *Charisius fasciatus* differ from those of *Charisius picturatus* (Fig. 4) by having the yellow regions of the elytral bands (Figs 1–3) less irregular and the basal band larger, extending to near the sutural margin. Some of the specimens from San Cristóbal are virtually indistinguishable in color from those of *Charisius mexicanus*. They may be distinguished only by dissection of the male eighth sternal lobes and by the distinctly finer punctation of the pronotum. Adults of *Charisius granulatus* closely resemble the other species of the fasciatus group in the color patterns, however adults of this species may be easily distinguished from all other species of this group by the more coarsely punctate pronotum, by having the surface between the punctures coarsely granulate and not shining and by the narrow apicale having the sides distinctly sinuate before the apex (Fig. 25).

I have provisionally assigned one specimen from El Salvador to this species, but it differs in having the apical two yellow bands reduced to small spots and in having the elytral striae completely unimpressed between the strial punctures on the apical fourth of the elytra. No other differences were noted.

Adults were collected by beating dead leaves in a pine-oak forest, from a Malaise trap set in an oak forest and from dead leaves alongside a logging road in the Sierra de las Minas, Guatemala. I reared adults from larvae and pupae collected from wood in hollow logs and from dead wood found in a tree hole.

#### 2. Charisius picturatus

Champion

http://species-id.net/wiki/Charisius_picturatus

Fig. 4

Charisius picturatus Champion (1893: 565, pl, 23, fig. 21); Campbell (1965: 48, Figs 10, 17).

##### Type.

Lectotype, male, Omilteme, Guerrero, Mexico 8000 ft (Campbell 1965: 48). The specimen is in the BMNH.

##### Distribution and records.

This species was described from one male and one female from Omiltemi, Guerrero, Mexico. I have provisionally assigned two additional females to this species.

MEXICO: Mexico: San Cayetana, Villa de Allende, 16.IX.1953, V Aquilar Vegs. (CNCI) 1. Oaxaca: Route 190, 33 mi NW Oaxaca, 4–5.IX.1967, G Ball, TL Erwin, RE Leech (CNCI) 1.

##### Remarks.

These specimens agree in all respects with those previously described by Champion except that the overall coloration is a darker reddish-brown. The specimen from Oaxaca was collected from an oak forest.

#### 3. Charisius mexicanus

Campbell

http://species-id.net/wiki/Charisius_mexicanus

Figs 5 , 14

Charisius mexicanus Campbell (1965: 49, Figs 4, 8, 11, 18).

##### Type.

Holoype, male, 5.2 miles west Acultzingo (Veracruz), Puebla, Mexico (Campbell 1965: 49). The specimen is in the BMNH.

##### Distribution and records.

This species was previously known from Mexico in the states of Morelos and Puebla. The species has been collected at elevations ranging from 1800 to 2700 m. The following new records are cited.

MEXICO: Guerrero: 1.3 km SW Filo de Caballo, 2700 m, 17.VII.1992, RS Anderson (CMNC) 1; 3 mi SW Filo de Caballo, 17.VII.1984, Carroll, Schaffner, Friedlander (EGRC) 3; 18-20 km SW Filo de Caballo, 9000 ft, 17.IX.1989, E Giesbert & JE Wappes (JEWC) 2. Mexico: San Cayetano, Villa de Allende, 16.IX.1953, V. Aguilar Vegs. (JMCC) 1; 3.6 km W Villa Victoria, 1925/10002, 2,530 m, 15–16.VII.1979, EL & KW Sleeper (CASC) 1. Michoacan:: Cerro Estimbo, 15.9 km NW Quiraga, 1943/10148, 13-14.VII.1979, 2,225 m, EL & KW Sleeper (CASC) 1. Morelos: Tres Marías, Wickham (USNM) 3. Oaxaca: El Cerazal, 16 km NE Oaxaca, 2300 m, 12.VI1979, H & A Howden (CMNC) 2; 20 mi S Juchatengo, Route 131, 6,000 ft, HF Howden (JMCC) 1; Hwy 131, 70 km S Oaxaca, 2150 m, 17.VI.1979, H &A Howden (CMNC) 1; 14 km N San Juan del Estado, 2600 m, 4.VIII.1986, H & A Howden (CMNC, JMCC) 2; Hwy. 175, 3 mi N Suchixtepec, 9500 ft, 4.VI.1971, DE Bright (CNCI) 1; 8 km S Suchixtepec, 6.VIII.1986, H & A Howden (CMNC, JMCC) 4.

##### Remarks.

There is little variation in the color pattern of this species (see Fig. 5). As previously mentioned, males of *Charisius fasciatus* from the state of Chiapas in Mexico may be distinguished from those of *Charisius mexicanus* with certainty only by examination of the male eighth sternal lobes. The adults of *Charisius mexicanus* can usually be distinguished by the distinctly coarser punctation of the pronotum (Fig. 14) and by the shorter apicale of the aedeagus (apicale 2.5–2.9 as long as basale).

Most adults were collected by beating dead leaves of oak trees. The specimens collected by EL Sleeper were taken in a boreal forest and an oak-pine woodland. All known records of *Charisius mexicanus* are from north of the Isthmus of Tehuantepec and those of *Charisius fasciatus* are from south of the Isthmus.

This species was previously known only from the highlands of central Mexico. These are the first records of the species from the Mexican states of Guerrero and Oaxaca.

#### 4. Charisius granulatus sp. n.

http://zoobank.org/9DA0E160-6B50-440E-ACAF-BBDB641D6F5E

http://species-id.net/wiki/Charisius_granulatus

Figs 6 , 15

##### Description.

Dark reddish-brown; elytra yellow with broad basal, large medial, and smaller V-shaped piceous markings (Fig. 6). Length 9.9–10.9 mm.

Head coarsely, densely, evenly punctate; punctures separated by average distance less than diameter of a puncture. Eyes moderate in size, mean ocular index of 5 specimens 35.4 (30–40).

Pronotum distinctly wider than long, mean pronotal index of 5 specimens 82.6, ranging from 81 to 84; surface microsculpture densely, coarsely granulate, opaque; punctures coarse (Fig. 15), moderately dense, separated on center of disc by average distance equal to or slightly greater than diameter of a puncture, punctures becoming finer and distinctly more widely separated on sides of disc; sides straight, subparallel from base to apical fourth then convexly narrowed to apex; transverse groove broad, moderately deeply impressed, disc shallowly, but distinctly impressed along midline. Prosternum and hypomeron with a few widely scattered, moderately coarse punctures. Metaventrite normally elongate, length between meso- and metacoxae distinctly longer than length of mesocoxal cavity; surface finely, moderately sparsely punctate medially, punctures becoming coarser approaching sides. Ventrites with punctures fine; last two ventrites slightly more coarsely and densely punctate. Elytra with striae moderately impressed basally, becoming deeply impressed towards apex; strial interstices convex.

Male. Anterior tibiae not sexually modified; anterior tarsal claws each with 7–9 teeth. Ventrite five not impressed medially. Lobes of eighth sternum (Fig. 21) broad, slightly curved medially, apices broadly, evenly rounded; apical and inner margins with row of very fine, short, dentiform setae; viewed laterally, lobes only slightly deflexed. Lobes of ninth sternum short, moderately broad, with apices evenly convex. Aedeagus with apicale (Fig. 25) narrow, with sides narrowed from base to narrowly rounded apex; sides moderately strongly sinuate just before apex.

Female. Anterior tarsal claws each with 7 teeth. Elytra with sutural margin and apex entire.

##### Types.

Holotype, male, with labels as follows: GUAT., Zac., 8 km N San Lorenzo, 10.VI.1993, 6700’, J. M.Campbell/ HOLOTYPE _ Charisius granulatus, desig. 2013, J.M.Campbell. The specimen is deposited in the CNCI.

Paratypes: two males and two females deposited in the collections of JEWC and JMCC.

##### Distribution and records.

This species is known only from the Departamentos of El Progresso and Zacapa in Guatemala.

GUATEMALA: El Progresso: above Los Albores, 8000 ft, 7–8.V.1991, E Giesbert (JMCC, JEWC) 2; 28–29 km N San Augustin, 7–8500 ft, 19–21.IV.1990, J. E. Wappes (JMCC, JEWC) 2. Zacapa: 8 km N San Lorenzo, 10.VI.1993, 6700 ft, JMC (CNCI) 1.

##### Etymology.

The species name granulatus is derived from the unique granulate microsculpture on the pronotal disc.

##### Remarks.

Adults of the species *Charisius granulatus* may be easily distinguished from those of all other species of *Charisius* in having the pronotum moderately densely, coarsely punctate with the surface between the punctures coarsely, densely granulate, opaque. The color pattern of the elytra (Fig. 6) will readily distinguish adults of *Charisius granulatus* from those of *Charisius mexicanus* and the Chiapas population of *Charisius fasciatus*. Males differ from those of all other species of the fasciatus group in lacking any trace of an expansion on the inner surface of the anterior tibiae.

### Subalatus Group

Species of this group may be easily distinguished from those of other species groups of *Charisius* by having the wings greatly reduced, shorter than the elytra and the functionally related shortening of the metaventrite (Fig. 18). In addition, the species are moderately large in size (7.6–9.8 mm in length), the male anterior tibiae are slightly, convexly widened on the inner margin, and the male fifth visible ventrite is not impressed medially. Males of *Charisius apterus* are unknown.

This species group contains two flightless species, one *Charisius subalatus*, formerly placed in the genus *Narses*, and the new species *Charisius apterus*.

#### 5. Charisius apterus sp. n.

http://zoobank.org/C6B07DD8-B980-4E47-9D7E-D2AE5FC1F67C

http://species-id.net/wiki/Charisius_apterus

Figs 7 , 20

##### Description.

Dark reddish-brown; elytron yellow with extensive brunneous markings as follows: moderately broad, transverse bands across basal fourth and across middle; narrow, jagged band across apical fourth and extreme apical portion; suture brunneous (Fig. 7). Length 9.8 mm.

Head coarsely, densely punctate, punctures separated by distance distinctly less than diameter of a puncture. Eyes small, widely separated dorsally, ocular index of female holotype 47. Pronotum slightly wider than long, PI of holotype 88; surface with microsculpture finely, uniformly granulate, not shining; moderately densely and coarsely punctate, punctures separated by distance slightly greater than diameter of a puncture; punctures evenly distributed except becoming sparser on narrow band at anterior lateral angles of pronotum; sides distinctly convex, widest at apical third, distinctly narrowed towards base; transverse groove broad, shallowly impressed; midline broadly, shallowly impressed on basal half.

Prosternum and hypomeron sparsely, finely punctate; postcoxal extension of sides of pronotum with row of coarse, moderately shallow impressions. Metaventrite short, length between meso- and metacoxae subequal in length to length of mesocoxal cavity; moderately coarsely and densely, evenly punctate. Abdomen with basal three ventrites with a few scattered, fine punctures; apical two ventrites more densely punctate with punctures separated by distance approximately two to three times diameter of puncture. Elytra 3.2 times longer than pronotum (in unique holotype) with striae shallowly, evenly impressed throughout; strial punctures coarse, narrowly separated along striae; intervals slightly convex. Wings reduced, flightless.

Male. Unknown.

Female. Anterior tarsal claws each with 6 teeth. Elytra with sutural margin near apex and apex emarginate as in Fig. 20. Fifth visible sternite broadly, shallowly impressed medially.

##### Type.

Holotype, female with labels as follows: MEX. OAXACA, 2 mi. S Cerro Pelon, 03 JUL 1982, 8-9000 ft. MA Ivie colr./ Holotype _, Charisius apterus, desig. 2013, JM Campbell. The holotype is deposited in the collection of the University of Montana.

##### Distribution and records.

*Charisius apterus* is known only from the type locality in Oaxaca, Mexico.

##### Etymology.

The species name apterus is derived from the species having the flight wings reduced to short stubs.

##### Remarks.

Adults of this remarkable flightless species can be easily distinguished from those of all other species of *Charisius* except *Charisius subalatus* by having the wings shorter than the elytra and the associated reduction in length of the metaventrite. The female holotype differs from specimens of *Charisius subalatus* by the distinctive elytral markings (Fig. 7) and by the unusual modifications of the elytral apices (Fig. 20). Other than the reduced wings and modified elytral apices of the female, the species is readily assigned to *Charisius* by the fact that the body is completely glabrous dorsally, the pronotum has a distinct, transverse depression across the base, and the antennae are elongate with the apical segments at least 2 times longer than wide.

#### 6. Charisius subalatus

(Champion) comb. n.

http://species-id.net/wiki/Charisius_subalatus

Figs 8 , 18 , 22 , 26

Narses subalatus Champion (1888: 424, pl. 19, Figs 15, 16a, 16b).

##### Description.

Uniformly reddish-brown; without elytral markings (Fig. 8). Length 7.6–8.7 mm.

Head coarsely, densely, evenly punctate on vertex; punctures separated by average distance less than diameter of a puncture. Eyes moderate in size, widely separated dorsally; mean OI of 9 specimens 45.2 (range 42–48). Pronotum slightly wider than long, average PI of 9 specimens 90.5 (range 87–96); with surface very finely granulate, opaque; finely, shallowly, sparsely punctate, punctures separated by average distance at least two times diameter of a puncture; punctures evenly distributed over most of disc except becoming nearly impunctate near sides; sides distinctly curved, widest across middle; with midline shallowly impressed in basal half. Prosternum and hypomeron with a few widely scattered, moderately coarse punctures; postcoxal extension of sides of pronotum with a few coarse, moderately shallow impressions. Metaventrite (Fig. 18) short, length between meso- and metacoxae shorter than length of mesocoxal cavity; moderately coarsely, densely, contiguously punctate medially in males, punctures becoming coarser and sparser approaching sides; coarsely punctate medially in female with punctures distinctly separated, punctures becoming coarser approaching sides. Ventrites finely, moderately sparsely punctate. Elytra 3.1–3.3 times longer than pronotum; striae shallowly, evenly impressed throughout (in specimens from Miramundo, Guatemala and El Salvador striae completely unimpressed between punctures); strial punctures coarse, narrowly separated along striae; intervals flat. Wings reduced, distinctly shorter than elytra; flightless.

Male. Anterior femora with patch of fine, dense pubescence on middle of ventral margin. Anterior tibiae slightly widened near middle on inner margin. Fifth ventrite unimpressed medially. Eighth sternal lobes (Fig. 22) broad, only slightly curved medially and distinctly curved ventrally near apices; apices of lobes narrowly rounded; viewed laterally, apices of lobes distinctly deflexed. Lobes of ninth sternum short, broad, with apices almost truncate. Aedeagus with apicale (Fig. 26) moderately narrow, with sides converging from base to near apex and distinctly constricted just before apex; apex narrowly rounded; viewed laterally, apicale slightly curved dorsally with apex very narrowly rounded; basale 2.8 to 2.9 times longer than apicale.

Female. Elytra with sutural margin and apex entire.

##### Type.

Not designated. The species was described by Champion from Totonicapán, 8,500–10,500 feet and the Quiché Mountains, 8,000 ft, both in Guatemala. The type series is in the BMNH.

##### Distribution and records.

The following records extend the known range of this species from Chiapas in southern Mexico to El Salvador.

EL SALVADOR: Chalatenango; El Pital, 13.1 km N San Ignacio, 2650 m, 28.VIII.1994, R Anderson (CMNC) 1.

GUATEMALA: Jalapa: Miramundo, 8400 ft, 3.VII.1986, JMC (CNCI, JMCC) 3. Quetzaltenango: Balneario Georginas, 1920–2460 m, 19.VI.1993, F. Genier (CMNC) 2. San Marcos: Volcán Tacaná, SE slope, Rancho San Antonio, 9,000 ft, 27–28.VII.1972, GE Ball (CNCI) 1.

##### Remarks.

*Charisius subalatus* and *Charisius apterus* are the only known flightless species of the genus. Adults of *Charisius subalatus* may be easily distinguished from those of *Charisius apterus* by the lack of markings on the elytra, by the much finer and sparser punctation of the head and pronotum, and by the lack of emarginations on the suture and apex of the female elytra. The close similarity of the male terminalia of *Charisius subalatus*, particularly with the species of the fasciatus group, provides additional confirmation for placing this genus in synonymy with the genus *Charisius*.

This species is known only from high elevations. It has been collected from bromeliads, from a Berlese sample of leaf litter in a cloud forest, and by beating herbaceous vegetation along the edge of a forest.

### Zunilensis Group

Species of this group (Figs 9, 10) are distinguished by lacking any trace of elytral markings, by having the wings fully developed, in lacking any trace of a swelling on the inner margin of the male protibia, in lacking impressions on the male fifth visible ventrite, and in having the apicale of the aedeagus elongate.

This species group contains two species, one of which, *Charisius howdenorum*, is described as new. One species, *Charisius interstitialis* Champion, is placed in synonymy.

#### 7. Charisius zunilensis

Champion

http://species-id.net/wiki/Charisius_zunilensis

Fig. 9

Charisius zunilensis Champion (1888: p. 422, pl. 19, fig. 14); Campbell (1965: p. 50, Figs 6, 12).
Charisius interstitialis Champion (1888: p. 422); Campbell (1965: p. 50) [Syn. n.].
Charisius floridanus Linell (1901: p. 184); Campbell (1965: p. 51).

##### Types.

Lectotype, male, Cerro Zunil, 4,000–5000 feet, Guatemala (Campbell 1965, p. 50). The type of *Charisius interstitialis* is a lectotype, male, from Jalapa, Mexico. These lectotypes are in the collection of the BMNH. The holotype of *Charisius floridanus* is type 4174 in the USNM.

##### Distribution and records.

The species is now known from the Mexican state of Veracruz south to Honduras. It is common and widely distributed in the highlands of Guatemala and southern Mexico. The species has been collected at elevations ranging from 1500 to 2500 m.

GUATEMALA: Baja Verapaz: km 4.1 Chilasco Rd., 4.VI.1993, JMC (JMCC) 1; 6.5 km W Chilasco, 1600 m, 22.V.1991, R Anderson (CMNC) 6; 6.5 km W Chilasco, 19.VI.1993, 1800 m, JMC (JMCC) 2; 7.8 km W Chilasco, 1700 m, 24.V.1991, H & A Howden (CMNC) 2; 8.6 km W Chilasco, 1500 m, 24.V.1991, R Anderson (CMNC) 3; 8 km S Purulhá, 1600 m, 25 & 29.V.1991, H & A Howden (CMNC) 2; 127.6 km S Purulhá, 1500 m, 21.V.1991, R Anderson (CMNC) 1; border of departments of Chimaltenango and Sololá, near Los Robles, 6000 ft, 12.IX.1965, JMC (JMCC) 1. Guatemala: Guatemala City, Cerro Alux, 2200 m, 9.VI.1991, R Anderson (CMNC) 1. Quezaltenango: Santa María, 18.V.1966, 5,000 ft, JMC (JMCC) 1; 2 km N Santa María, near tunnel, 5500 ft, 10.VII.1965, 25–27, VIII.1965, 24.X.1965, JMC (CNCI, JMCC) 5; Volcán de Chicabal, 2100 m, 25.VIII.1965, JMC (CNCI) 1; 5.4 km SE Zunil, 2200 m, 19.VI.1993, R Anderson (CMNC) 1. Sacatepequez: Finca Florencia, 24.VI.1993, JMC (JMCC) 1. San Marcos: 20 km. W San Marcos, 2200 m, 3.X.1965, JMC (JMCC) 2; 8 km NE San Rafael Pie de la Cuesta, 2000 m, 4.VI.1966, JMC (CNCI, JMCC) 2. Suchitepequez: 5 km S Santiago Atitlan, 1500 m, 29.VIII.1965, JMC (JMCC) 1; Zunilito, 2 km N Finca Colimas, 6000 ft, 28.IV.1966, 6.V.1966, JMC (CNCI, JMCC) 7; 10 km NE Yepocapa, 8000 ft, 29.V.1966, JMC (JMCC) 1. Zacapa: San Lorenzo, Sierra de las Minas, 1740 m, 9–17.VII.1986, JMC (CNCI) 17; same locality, 7.VII.1986, L LeSage (CNCI) 1; same locality, 17–18.VI.1993, JMC (CNCI, JMCC) 7; 3 km NE San Lorenzo, Sierra de las Minas, 1800 m, 6.VII.1986, JMC (CNCI) 7; 5–8 km N San Lorenzo, 1900–2000 m, 10.VI.1993, H & A Howden (CMNC) 1; 5 mi N San Lorenzo, 12.VII.1986, JMC (JMCC) 3; 6 km N Lorenzo, 17.VI.1983, 6500 ft, JMC (CNCI, JMC) 6; 8 km NE San Lorenzo, 2100 m, 18.VII.1986, JMC (CNCI) 3; 8 km N San Lorenzo, 10.VI.1993, 6700 ft, JMC (CNCI, JMCC) 11.

HONDURAS: Francisco Morazán: 30 km E Tegucigalpa, Cerro Uyuca, 1800 m, 19.V.1994, H & A Howden (CMNC) 3; La Tigra Nat. Pk., NE Tegucigalpa, 1900 m, 4.VI.1994, H & A Howden (CMNC) 2; 10 km W Zamorano, Cerro Uyuca, 1950 m, 18.VIII.-2.IX.1994, S & J Peck (CMNC) 2.

MEXICO: Chiapas: 20 km N Bochil, Yerba Buena, 5700 ft, 8.VI.1969, JMC (CNCI) 1; 5 mi SW El Bosque, 4.VII.1969, Campbell & Bright (CNCI) 1; San Cristobal de las Casas, 17.V.1990, H & A Howden (CMNC) 1; 5 mi W San Cristobal, 19.V.1969, JMC (CNCI) 1; 8 mi NE San Cristobal, Tinijapa, 18.V.1969, 26.V.1969, JMC (CNCI, JMCC) 16; 8 mi NE San Cristobal, 28.VI.1969, JMC (CNCI) 4; 11 mi NE San Cristobal, 8.V.1969, HF Howden (CMNC) 1; 15 km SE San Cristobal, 11.VI.1989, H Howden (CMNC) 1. Veracruz: 7 km E Huatusco, 22.VI-2.VII.1983, 1250 m, S Peck (CMNC) 1; Jalapa, JT Mason (AMNH) 1.

##### Remarks.

*Charisius zunilensis* was previously known from only a few specimens collected on the Cerro Zunil near Quetzaltenango, Guatemala and *Charisius interstitialis* was known only from a few specimens collected at Jalapa, Veracruz, Mexico. In my previous revision of the genus (1965), I suggested that the two species could by synonyms, but delayed placing one of them in synonymy until additional material was available. Based on the new records cited below, there is little doubt that only one species is at hand.

There is considerable variation in the development of the microsculpture on the head and pronotum of this species ranging from almost completely lacking to moderately coarsely and densely granulate, and in the density and coarseness of punctation between the eyes and on the center of the pronotum. However, all degrees of variation can be found in specimens from throughout the range so there is no justification for recognizing more than one species. Most adults of this species were collected by beating dead leaves, particularly of oak trees and coffee shade trees.

#### 8. Charisius howdenorum sp. n.

http://zoobank.org/771CBAF4-8CED-41A2-B9FD-62783030C62D

http://species-id.net/wiki/Charisius_howdenorum

Figs 10 , 19 , 23 , 27

##### Description.

Dark reddish-brown to dark brown (Fig. 10); antennae and legs slightly paler than body; elytra without markings. Length 6.5–8.6 mm.

Head moderately coarsely, densely, evenly punctate on vertex; punctures separated by average distance less than diameter of a puncture. Eyes moderate in size, mean OI of 10 specimens 43.9 (range 41–47). Pronotum distinctly wider than long, mean PI of 10 specimens 82.6, ranging from 77 to 86; surface with microsculpture moderately coarse, granulate, only slightly shining; punctures moderately coarse, moderately dense, separated on center of disc by average distance equal to or slightly greater than diameter of a puncture, punctures finer, distinctly sparser on sides; sides straight or slightly sinuate on basal two-thirds, then evenly, convexly narrowed to apex; convex, widest near middle then evenly narrowed to base and apex; disc often faintly impressed along midline. Prosternum and hypomeron moderately sparsely, coarsely, irregularly punctate; punctures separated by distance greater than diameter of a puncture. Metaventrite finely, moderately densely punctate medially in male; punctures becoming coarser approaching sides; finely and sparsely punctate medially in female. Abdomen with fine, scattered punctures on basal three ventrites, last two visible ventrites more coarsely and slightly more densely punctate. Elytra (Fig. 19) with striae unimpressed; strial interstices flat.

Male. Anterior tibia not widened on inner side. Fifth ventrite unimpressed medially. Lobes of eighth sternum (Fig. 23) broad, straight, apices evenly convex; viewed laterally, lobes slightly deflexed apically. Lobes of ninth sternum short, moderately broad, with apices broadly convex. Aedeagus with apicale (Fig. 27) moderately narrow; sides narrowed from base to near middle then subparallel to evenly convex apex; viewed laterally, apicale straight, basale 3.2–3.3 times longer than apicale.

Female. Elytra with sutural margin and apex entire.

##### Types.

Holotype, male, with labels as follows: MEX., Tinijapa, 8 km NE San Cristobal, Chis., V.26.1969, J. M. Campbell/ HOLOTYPE _, Charisius howdenorum, desig. 2013, J.M.Campbell. The specimen is deposited in the CNCI.

Paratypes, 23 in the CNCI the CMNC and JMCC.

MEXICO: Chiapas: 5 mi W San Cristobal de las Casas, 19.V.1969, JMC (CNCI) 1; 8 mi NE San Cristobal, 26–28.VI.1969, JMC (CNCI, JMCC) 5; 11 mi NE San Cristobal, 18.V.1969, HF Howden (CMNC) 1; Tinijapa, 8 mi NE San Cristobal, 26.V.1969 JMC (CNCI, JMCC) 11; nr. Tinijapa, 8 km NE San Cristobal, 18.V.1969, JMC (CMNC, JMCC) 6.

##### Etymology.

This species is named in honor of Dr. Henry and Anne Howden, Canadian Museum of Nature, Alymer, Quebec, Canada who have facilitated several of my trips to Mexico and Guatemala; and have collected many of the specimens described in this paper.

##### Remarks.

Adults of *Charisius howdenorum* are similar to those of *Charisius zunilensis*, but differ most noticeably in having the elytral striae unimpressed between the strial punctures (Fig. 19). They also differ in being somewhat darker in color, in having the pronotum less shining with the microsculpture distinctly more coarsely and densely granulate, and, in the males, the metaventrite is finely, but distinctly more densely punctate medially. There is little difference between the male terminalia of the two species.

### Salvini Group

Species of this group may be easily distinguished by having the wings fully developed, in having at least the apex of the elytra piceous to black, in being moderately large in size (length 7.9–10.9 mm), in having the male anterior tibiae distinctly widened on the inner margin, in having the male fifth ventral segment deeply impressed medially, and the short apicale of the aedeagus (Fig. 28).

The species group contains two species, one of which is described as new. *Charisius salvini* differs from *Charisius punctatus* and all other species of the genus in having only the two basal segments of the male protarsus lobed ventrally.

#### 9. Charisius salvini

Champion

http://species-id.net/wiki/Charisius_salvini

Figs 11 , 16

Charisius salvini Champion (1888: p. 423, pl. 19, fig. 15); Campbell (1965: 52, Figs 5, 14).

##### Type.

Lectotype, male, Calderas, Guatemala (Campbell 1965: 52). The specimen is in the BMNH.

##### Distribution and Records.

*Charisius salvini* was previously known from the highlands of southeastern and southcentral Guatemala. The species is now known to be widespread in Guatemala and is reported from El Salvador, Honduras and Nicaragua at elevations between 1340 and 1830 meters in elevation.

EL SALVADOR: Cerro Verde, 2000 m, 1.V. and 4.V.1971, HF Howden (CMNC, JMCC) 3.

GUATEMALA: Baja Verapaz: 5 km above Ixpaco, 4300 ft, 22.VI.1983, JMC (CNCI) 1; 9 km S San Jeronimo, 4500 ft, 25.VI.1966(JMCC)2; 7.8 mi W Chilasco, 1700 m, 24.V.1991, H & A Howden (CMNC) 1. Chimaltenango: 7 km N Laguna de Calderas, 14.V.1966, JMC (CNCI, JMCC) 5. Esquintla: 3 km E San Vicente Pacayá, 5500 ft, 14.V.1966, JMC (CNCI, JMCC) 5; 4 km N Palin, 4500 ft, 21.VI.1966, JMC (JMCC) 1. Guatemala: Cerro Alux, 24.VI.1993, RS Anderson (CMNC) 1; 14.5 km SE Guatemala, Puenta Parada, 1790 m, 13.VI.1991, A Howden (CMNC, JMCC) 3. Sacatepequez: Antigua, IX.1959, NLH Krauss (USNM) 1; Finca Florencia, 24.VI.1993, JMC (CNCI, JMCC) 4; 1 km W Sta Lucia Milpas Altas, 3.VI.1993, JMC (CNCI) 1. Suchitepéquez: 2 km N Finca Colima, Zunilito, 6000 ft, 6.V.1966, JMC (JMCC) 1. Quezaltenango: 12.9 km SW Zunil, 1340 m, 18.VI.1993, RS Anderson (CMNC) 1.

HONDURAS: Cortez: 25 km N Cofradia, PN Cusucol, 1550 m, 19.IX–7.X.1994, S & J Peck (CMNC) 1. Francisco Morazán: 30 km E Tegucigalpa, Cerro Uyuca, 1800 m, 3.VI.1994, H & A Howden (CMNC) 1. Ocotepeque: 11 km E Ocotepeque, 1450 m, 16.VI.1994, R Anderson (CMNC) 1.

NICARAGUA: Cerro Chimborazo, 13°02'N, 85°56'W, 20.XI.1971, H Stockwell (CNCI) 1.

##### Remarks.

Specimens of *Charisius salvini* may be distinguished from those of all other species of *Charisius* by being uniformly reddish-brown dorsally with only the extreme apex of the elytra piceous to black and in having only the basal two segments of the male protarsus lobed ventrally. They also differ from all other species except the following in having the pronotum coarsely, moderately densely, evenly punctate and by the very distinctive shape of the male eighth sternal lobes and aedeagus (see Campbell 1965, Fig. 5).

I have provisionally assigned one male from Nicaragua to this species. It agrees well with all the characters of *Charisius salvini* except the male anterior tibiae are slightly sinuate on the inner margin and it has a piceous, transverse band acoss the apical third of the elytra. This record extends the known range of species of *Charisius* south to Nicaragua.

Most specimens were collected by beating clumps of dead branches and leaves in shrubs and low hanging trees. Adults were collected from coffee shade trees near the upper limits of coffee growing zones.

#### 10. Charisius punctatus sp. n.

http://zoobank.org/27045083-198A-4614-B95A-7786EDDA7842

http://species-id.net/wiki/Charisius_punctatus

Figs 12 , 24 , 28

##### Description.

Body reddish-brown; elytra testaceus with each elytron having piceous markings as follows (Fig. 12): a small circle near middle, a narrow, crescent-shaped band at apical fourth and extreme apex piceous to black (Fig. 12). Length 7.0–10.9 mm.

Head moderately coarsely punctate on vertex; punctures separated by distance subequal to diameter of a puncture. Eyes moderately small, mean OI of 5 males 32.7 (range 30–35) and of five females 38.6 (range 37–40). Pronotum distinctly wider than long, mean PI of 11 specimens 84.6 (range 83 to 88); surface smooth, shining; punctures coarse, moderately densely, evenly distributed, separated by average distance distinctly greater than diameter of a puncture; sides sinuate near base, widest near middle than evenly convex to just before apex; disc evenly convex in cross section. Prosternum and hypomeron coarsely, densely punctate; punctures separated by distance less than diameter of a puncture. Metaventrite finely, sparsely punctate medially, punctures becoming coarser and denser approaching sides. Ventrites with punctures moderately fine, sparsely distributed; last two visible ventrites more coarsely and densely punctate. Elytra with striae moderately deeply, evenly impressed throughout; strial interstices moderately convex.

Male. Anterior tibia triangularly widened on inner side near middle. Fifth ventrite broadly, deeply, triangularly impressed medially. Lobes of eighth sternum (Fig. 24) broad, with apical third broadly expanded, apices obliquely truncate; viewed laterally, distinctly deflexed apically. Lobes of ninth sternum short, moderately broad, with apices evenly convex. Aedeagus with apicale (Fig. 28) short, moderately narrow; sides narrowed from base to narrowly, evenly convex apex, slightly constricted medially; viewed laterally, apicale strongly curved dorsally; basale 7.1–7.9 times longer than apicale.

Female. Elytra with sutural margin and apex entire.

##### Type.

Holotype, male, with labels as follows: GUAT. Depto. Zacapa, San Lorenzo, 1740 m, Sierra de las Minas, 18.VII.1986, J.M.Campbell/ beating mixed vegetation in pine-oak forest/ HOLOTYPE _, Charisius punctatus, desig. 2013, JM Campbell. The specimen is deposited in the CNCI.

Paratypes, 44 in the CNCI, CMNC, JMCC, and the JEWC.

##### Distribution and records.

This species is known only from Guatemala between 1400 and 1740 meters in elevation.

GUATEMALA: Alta Verapaz: Jnct. Rds. Coban & Purulhá, 5500 ft, 24.VI.1966, JMC (JMCC) 1. Baja Verapaz: Pantin Rd., 9 km N Salamá, 20–21.VI.1993, JMC (JMCC) 1; 6–9 km E Purulhá, 5000 ft, 15–24.IV.1990, E Geisbert (JEWC) 1. Chimaltenango: Yepocapa, 4600 ft, 10.V.1966, JMC (JMCC) 1. Zacapa: San Lorenzo, Sierra de las Minas, 1740 m, 8–11.1986, JMC (CNCI, JMCC) 10; same locality, 11.VII. and 17.VII.1986, L LeSage (CNCI) 2; same locality, 5200 ft, 17–19.VI.1986, JMC (CNCI, JMCC) 11; nr. San Lorenzo, 13.IV.1990, JE.Wappes (JEWC) 1; vic. San Lorenzo, 5800 ft, 10–15.VI.1991, E Geisbert (JEWC) 1; 13 km N Hwy, San Lorenzo Rd., 3000 ft, 17.VI.1993, JMC (CNCI) 1.

##### Etymology.

The species name punctatus refers to the coarse punctation of the pronotum.

##### Remarks.

Adults of *Charisius punctatus* are very similar to those of *Charisius salvini*, but may be easily distinguished by the additional piceous markings on the elytra (Fig. 9). Males may also be distinguished by having the basal four segments of the anterior tarsi lobed ventrally, by having a triangular expansion on the inner margin of the anterior tibia; by the broader and deeper median impression of the fifth abdominal ventrite, and by the more broadly expanded apical portion of the eighth sternal lobes (Fig. 24) which are obliquely truncate.

The species has been collected beating mixed dead and herbaceous vegetation in a pine-oak forest.

### Key to species of *Charisius* Champion

**Table:** 

1	Wings reduced, flightless species, metaventrite short (Fig. 18)	2
–	Wings fully developed, metaventrite normal in length (Fig. 17)	3
2	Elytra with transverse yellow bands; apex of female elytron emarginate (Figs 7, 20)	5. *Charisius apterus* sp. n.
–	Elytra uniform in color (Fig. 8), apex of elytra in female entire	6. *Charisius subalatus* (Champion)
3	Elytra with distinct, yellow, transverse markings (Figs 1–6).	4
-	Elytra uniform in color or with piceous to black markings (Figs 9–12)	8
4	Pronotum with surface microsculpture densely, coarsely granulate, opaque; punctures coarse, moderately densely distributed (Fig. 15)	4. *Charisius granulatus* sp. n.
–	Pronotum with surface microsculpture fine, visible only under high magnfication (64×), shining; punctures fine, sparsely distributed (Figs 13, 14)	5
5	Elytra with basal yellow band greatly expanded, basal band interrupted only by elytral suture (Figs 1, 5–6)	6
-–	Elytra with basal yellow band reduced to oval spot or absent, not reaching suture (Figs 2, 3, 5)	7
6	Male eighth sternal lobes narrow (see Campbell 1965, p. 55), evenly curved medially; known from south of the Isthmus of Tehuantepec in Mexico	1. *Charisius fasciatus* Champion (in part)
–	Male eighth sternal lobes broad, (see Campbell 1965, p. 55) only slightly curved medially; known from north of the Isthmus of Tehuantepec of Mexico	3. *Charisius mexicanus* Campbell
7	Basal yellow spot extending from sides to middle of each elytron (Fig. 4); sides of pronotum parallel for basal half	2. *Charisius picturatus* Champion
–	Basal yellow spot of elytra either absent (Fig. 2) or represented by a large, oval spot placed in- middle of each elytral disc (Fig. 3)	1. *Charisius fasciatus* (in part)
8	Elytra with at least apex piceous to black (Figs 9, 11); last visible sternite of male deeply impressed medially	9
–	Elytra uniform in color (Figs 9, 10); last visible sternite of male not impressed	10
9	Elytra with only apex piceous to black (Fig. 11); male protarsus with only basal two segments lobed ventrally; male anterior tibiae at most slightly sinuate on internal margin	9. *Charisius salvini* Champion
–	Each elytron with a median piceous spot and a narrow, crescent-shaped band at apical fourth (Fig. 12); male protarsus with basal four segments lobed ventrally; male anterior tibiae with distinct, triangular swelling on inner margin	10. *Charisius punctatus* sp. n.
10	Elytra with striae distinctly impressed between strial punctures from base to apex (Fig. 9)	7. *Charisius zunilensis* Champion
–	Elytra with striae completely unimpressed between strial punctures (Fig. 19)	8. *Charisius howdenorum* sp. n.

## Conclusions

Species of *Charisius* are widely distributed at moderately high to high elevations from central Mexico south to Nicaragua. Elevations have been recorded from 1340 meters to 2800 meters. Species of the genus have several characters normally associated with higher elevations. All species of the genus are glabrous dorsally and two of the species (*Charisius apterus* and *Charisius subalatus*) have the flight wings reduced and are flightless.

The close similarity of the male eighth sternal lobes of *Charisius subalatus* with the species of the fasciatus group (compare Figs 21 and 22) are additional justification for placing *Narses* in synonymy with *Charisius* as previously suggested by Champion (1888, p. 423).

The species of the genus were previously unknown from south of Guatemala, however, additional collecting has extended their known range south to Nicaragua.

## Supplementary Material

XML Treatment for Charisius

XML Treatment for Charisius fasciatus

XML Treatment for Charisius picturatus

XML Treatment for Charisius mexicanus

XML Treatment for Charisius granulatus

XML Treatment for Charisius apterus

XML Treatment for Charisius subalatus

XML Treatment for Charisius zunilensis

XML Treatment for Charisius howdenorum

XML Treatment for Charisius salvini

XML Treatment for Charisius punctatus

## References

[B1] Campbell JM (1965) A revision of the Genus *Charisius* (Coleoptera: Alleculidae). The Coleopterists Bulletin 19: 43–56

[B2] Champion GC (1888) Family Cistelidae. In: Godman FD Salvin O (Eds) Biologia Centrali-Americana. Insecta Coleoptera. Vol. IV, pt. 1, 385–465, pls 17–21

[B3] Champion GC (1893) Heteromera. In: Godman FD Salvin O (Eds) Biologia Centrali-Americana. Vol. IV, pt. 1, 477–572

[B4] Lawrence JF Beutel RG Leschen RAB Ślipiński SA (2010) 2. Glossary of Morphological Terms. In: Leschen RAB Beutel RG Lawrence JH (Eds) Coleoptera, Beetles, Morphology and Systematics. Vol. 2: 9–20

[B5] Linell M (1901) Descriptions of some new species of North American heteromerous Coleoptera. Proceedings of the Entomological Society of Washington 4: 180–186

